# Inducible Lung Epithelial Resistance Requires Multisource Reactive Oxygen Species Generation To Protect against Viral Infections

**DOI:** 10.1128/mBio.00696-18

**Published:** 2018-05-15

**Authors:** Carson T. Kirkpatrick, Yongxing Wang, Miguel M. Leiva Juarez, Pooja Shivshankar, Jezreel Pantaleón García, Alexandria K. Plumer, Vikram V. Kulkarni, Hayden H. Ware, Fahad Gulraiz, Miguel A. Chavez Cavasos, Gabriela Martinez Zayes, Shradha Wali, Andrew P. Rice, Hongbing Liu, James M. Tour, William K. A. Sikkema, Ana S. Cruz Solbes, Keith A. Youker, Michael J. Tuvim, Burton F. Dickey, Scott E. Evans

**Affiliations:** a Department of Pulmonary Medicine, University of Texas MD Anderson Cancer Center, Houston, Texas, USA; b Tecnológico de Monterrey School of Medicine, Monterrey, Mexico; c The University of Texas Graduate School of Biomedical Sciences, Houston, Texas, USA; d Department of Molecular Virology and Microbiology, Baylor College of Medicine, Houston, Texas, USA; e Smalley Institute for Nanoscale Science and Technology, Rice University, Houston, Texas, USA; f Michael E. Debakey Heart and Vascular Institute, Houston Methodist Hospital, Houston, Texas, USA; Brown University

**Keywords:** inducible resistance, Toll-like receptors, lung epithelium, mucosal immunity, reactive oxygen species, viral pneumonia

## Abstract

Viral pneumonias cause profound worldwide morbidity, necessitating novel strategies to prevent and treat these potentially lethal infections. Stimulation of intrinsic lung defenses via inhalation of synergistically acting Toll-like receptor (TLR) agonists protects mice broadly against pneumonia, including otherwise-lethal viral infections, providing a potential opportunity to mitigate infectious threats. As intact lung epithelial TLR signaling is required for the inducible resistance and as these cells are the principal targets of many respiratory viruses, the capacity of lung epithelial cells to be therapeutically manipulated to function as autonomous antiviral effectors was investigated. Our work revealed that mouse and human lung epithelial cells could be stimulated to generate robust antiviral responses that both reduce viral burden and enhance survival of isolated cells and intact animals. The antiviral protection required concurrent induction of epithelial reactive oxygen species (ROS) from both mitochondrial and dual oxidase sources, although neither type I interferon enrichment nor type I interferon signaling was required for the inducible protection. Taken together, these findings establish the sufficiency of lung epithelial cells to generate therapeutically inducible antiviral responses, reveal novel antiviral roles for ROS, provide mechanistic insights into inducible resistance, and may provide an opportunity to protect patients from viral pneumonia during periods of peak vulnerability.

## IMPORTANCE

Viruses are the most commonly identified causes of pneumonia and inflict unacceptable morbidity, despite currently available therapies. While lung epithelial cells are principal targets of respiratory viruses, they have also been recently shown to contribute importantly to therapeutically inducible antimicrobial responses. This work finds that lung cells can be stimulated to protect themselves against viral challenges, even in the absence of leukocytes, both reducing viral burden and improving survival. Further, it was found that the protection occurs via unexpected induction of reactive oxygen species (ROS) from spatially segregated sources without reliance on type I interferon signaling. Coordinated multisource ROS generation has not previously been described against viruses, nor has ROS generation been reported for epithelial cells against any pathogen. Thus, these findings extend the potential clinical applications for the strategy of inducible resistance to protect vulnerable people against viral infections and also provide new insights into the capacity of lung cells to protect against infections via novel ROS-dependent mechanisms.

## INTRODUCTION

Lower respiratory tract infections present a worldwide public health threat, exerting a tremendous mortality and health care resource burden (1–3). Of an estimated 450 million annual episodes of pneumonia, viral pathogens may cause as many as 200 million cases (4, 5). Further, viruses are the most frequently identified pathogens in community-acquired pneumonia requiring hospitalization among adults and children in the United States (6, 7). Seasonal influenza pneumonias alone often cause more than 40,000 deaths in the United States annually, despite long-established vaccination programs (8). Moreover, the history of 50 million worldwide influenza-related deaths during the pandemic of 1918–1919 (9) remains a cautionary reminder of the potential lethality of this pathogen.

We have reported that the lung’s mucosal defenses can be stimulated to protect mice against a wide array of otherwise lethal pneumonias, including those caused by influenza A viruses (10–14). This inducible resistance is achieved following a single inhaled treatment comprised of a synergistic combination of Toll-like receptor (TLR) agonists: a diacylated lipopeptide ligand for TLR2/6, Pam2CSK4, and a class C unmethylated 2′-deoxyribocytidine-phosphate-guanosine (CpG) ligand for TLR9, ODN M362 (here, Pam2-ODN) (10, 13–15).

Inducible resistance against pneumonia requires intact lung epithelial TLR signaling, whereas no individual leukocyte populations have been identified as essential to Pam2-ODN-enhanced pneumonia survival (14). Given the epithelial requirement for inducible antiviral resistance, we sought to determine whether epithelial cells were sufficient to act as autonomous antiviral effector cells of therapeutically inducible protection. We postulated that generating antiviral responses directly from the principal target of many respiratory viruses—the respiratory epithelium (16)—could be a highly efficacious strategy to reduce virus-induced host pathology.

We report that Pam2-ODN induces active antiviral responses from intact lungs and isolated lung epithelial cells that reduce viral burden, attenuate infectivity, and enhance survival. Moreover, this protection requires epithelial generation of reactive oxygen species (ROS) via dual mechanisms, providing meaningful insights into the mechanisms of the novel synergistic interactions observed between the TLR ligands.

## RESULTS

### Pam2-ODN-inducible resistance is associated with reduced lung viral burden.

**FIG 1:**
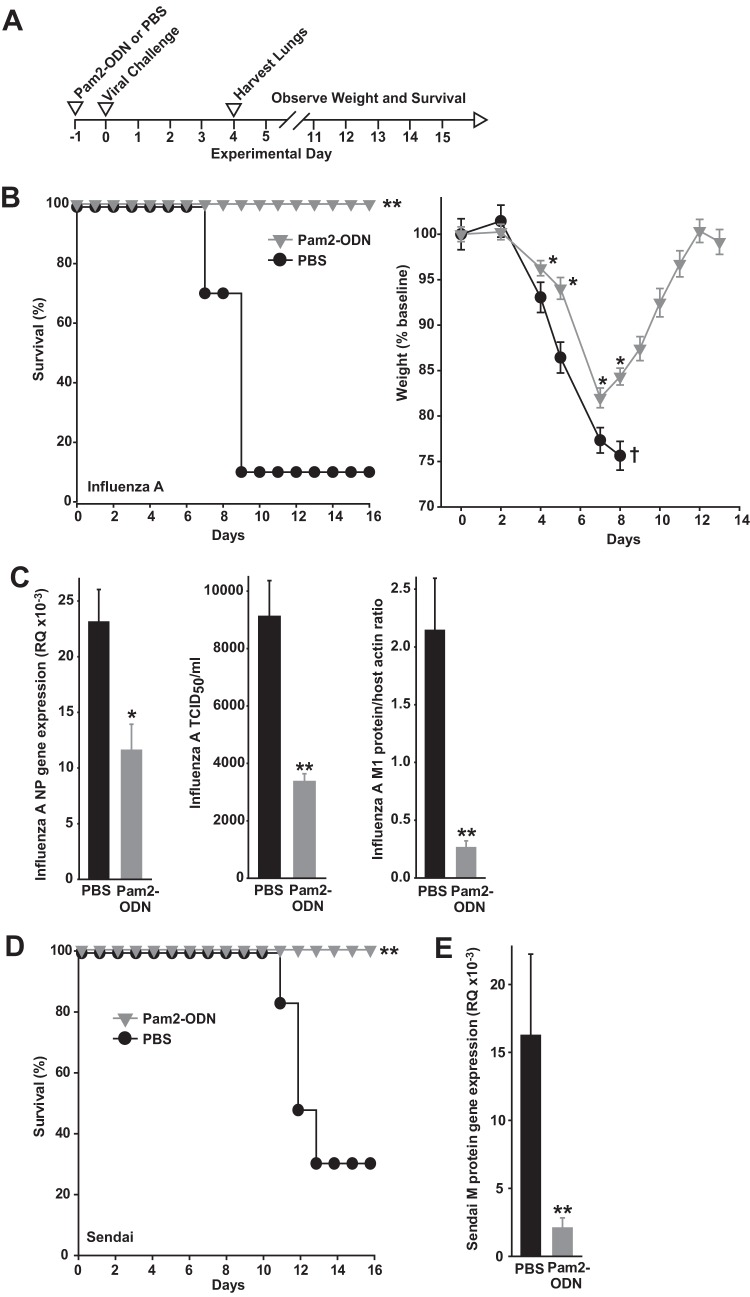
Protective Pam2-ODN treatment reduces lung virus burdens. (A) Schematic of challenges. (B) Survival (left) and weight (right) of C57BL/6J mice treated with PBS or Pam2-ODN 24 h prior to infection with influenza A virus. (C) Lung viral burdens of mice in panel A 4 days after infection, assessed by qPCR for expression of influenza A virus nucleoprotein (NP) gene relative to the host 18S gene (RQ) (left), hemagglutination (center), and immunoblot densitometry for viral M1 protein relative to host β-actin levels (right). PBS or Pam2-ODN was nebulized and administered to mice 24 h prior to infection with Sendai virus. (D and E) Survival (D) and Sendai virus (E) M gene expression in lung homogenates 4 days after infection. *n* = 15 mice/group in survival plots; *n* = 4 mice/group in viral burden experiments. *, *P* < 0.03 compared to PBS-treated group; **, *P* < 0.004 compared to PBS-treated group; †, only one mouse remained (summary statistic could not be calculated).

Although inducible protection against bacterial and fungal infections uniformly correlates with reductions in lung pathogen burden (12–14, 17, 18), the Pam2-ODN effect on lung viral burdens has not been assessed. Indeed, we found that the robust Pam2-ODN-induced protection against mouse-adapted influenza A (H3N2) virus pneumonia (Fig. 1A to C) is associated with significantly reduced lung virus burden, whether measured by viral gene expression, tissue infectivity, or viral protein (Fig. 1C). This effect was not restricted to influenza A virus or to orthomyxoviruses, as Pam2-ODN also protected against Sendai virus infection and reduced lung virus burden (Fig. 1D and E). Thus, Pam2-ODN-enhanced survival of infection correlates with reductions in pathogen burden for every tested infection model, including viruses. Moreover, the Pam2-ODN-induced lung viral burden differential continues to increase to the time of peak mortality (see Fig. S1A in the supplemental material), and inoculum titration studies (Fig. S1B) indicate that viral reductions of the magnitude observed in these studies are sufficient to afford profound survival increases.

10.1128/mBio.00696-18.1 FIG S1  Lung virus burden time course and correlation of viral inoculum and mouse survival. (A) Mice were infected with influenza A virus 24 h after treatment with PBS (sham) or Pam2-ODN, and lungs were harvested on the indicated day, homogenized, and submitted to immunoblotting for viral M2 protein. Results are densitometry ratios for viral M2:host glyceraldehyde 3-phosphate dehydrogenase (GAPDH). *n* = 3 to 4 mice/condition. *, *P* < 0.05 versus PBS-treated controls. ND, not detected. (B) Mice were infected with the indicated inocula of influenza A virus. Results shown are mouse survival 14 days after challenge. Red bars indicate inoculum intercepts at 80% and 20% survival. *n* = 5 to 7 mice/condition. Download FIG S1, EPS file, 2.3 MB. Copyright © 2018 Kirkpatrick et al. 2018 Kirkpatrick et al. This content is distributed under the terms of the Creative Commons Attribution 4.0 International license.

### Antiviral responses from isolated epithelial cells.

Although lung epithelial cell TLR signaling is required for Pam2-ODN-induced influenza virus protection *in vivo* (14), whether isolated lung epithelial cells are sufficient to generate autonomous antiviral responses to Pam2-ODN treatment was unknown. As the survival advantage in intact animals was associated with reduced lung viral burdens, we tested whether Pam2-ODN treatment of epithelial cells could restrict viruses *in vitro* without leukocyte contributions.

**FIG 2:**
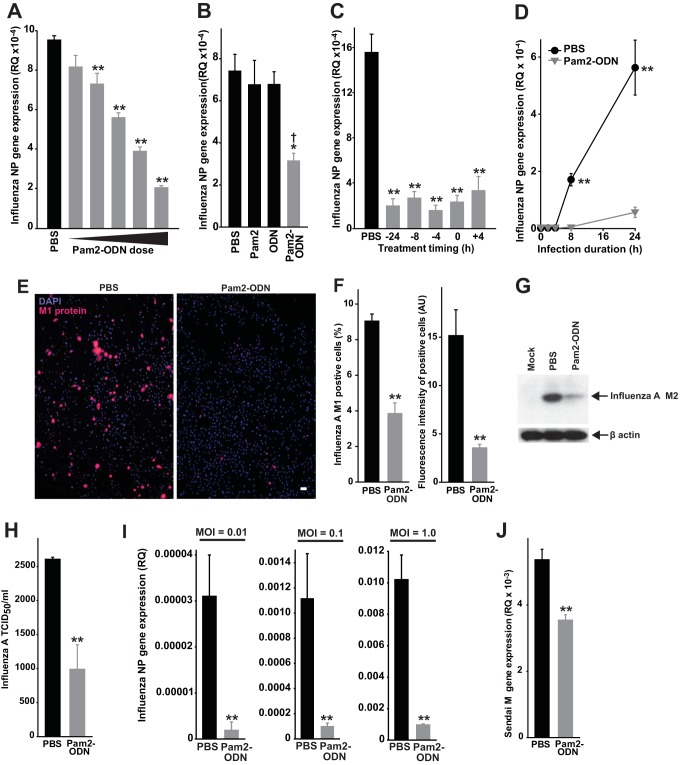
Pam2-ODN-induced viral burden reductions in isolated epithelial cells. (A) HBEC3kt cells were treated with PBS or escalating doses of Pam2-ODN for 4 h, and then infected with influenza A virus. Results shown are expression levels of influenza virus NP gene expression after 24 h. (B) NP gene expression in HBEC3kt cells 24 h after influenza virus infection following 4 h of treatment with the indicated agents. (C) HBEC3kt cells were treated with PBS or Pam2-ODN for the times indicated, and results are relative to those after infection with influenza A virus. Results shown are NP expression 24 h after infection. (D) NP gene expression in HBEC3kt cells at the indicated time points after infection. (E) HBEC3kt cells were treated for 4 h with PBS or Pam2-ODN and infected for 24 h with influenza A virus. Micrographs show merged DAPI and fluorescent anti-M1 antibody images. Scale bar = 50 µm. (F) Quantification of cells positive for M1 staining (left) and mean fluorescence intensity of M1-positive cells (right) from panel J. (G) Immunoblot for viral M2 protein in HBEC3kt cells treated for 4 h with PBS or Pam2-ODN and then infected for 24 h with influenza A virus. (H) Results (TCID_50_ per milliliter) for HBEC3kt cells treated with Pam2-ODN or PBS for 4 h and then infected with influenza A virus for 24 h. (I) NP gene expression of HBEC3kt cultures 24 h after infection with the indicated influenza A virus inocula. (J) Sendai virus M gene expression in HBEC3kt cells 24 h after infection. *n* = 5 samples/condition. *, *P* < 0.03 compared to PBS-treated group; **, *P* < 0.004 compared to PBS-treated group; †, *P* < 0.01 compared to either single-ligand treatment.

Figure 2A shows dose-dependent reductions in influenza virus burden of human-derived HBEC3kt lung epithelial cell cultures by Pam2-ODN over a dynamic antiviral range exceeding 2 log_10_ concentrations (shown in a fixed concentration ratio, from 0.03 µM ODN:0.12 µM Pam2 to 3.1 µM ODN:12.4 µM Pam2), confirming their sufficiency to effect protection *in vitro*. Viral loads were further reduced at higher Pam2-ODN concentrations (see Fig. S2 in the supplemental material), although epithelial exposure to such high local concentrations is not likely achieved following nebulization *in vivo*. In fact, based on estimation of Pam2-ODN deposition in 20 µl of airway lining fluid of a mouse (19) or in 10 to 30 ml of airway lining fluid in a human (20), *in vivo* epithelial exposure to Pam2-ODN, while likely much higher, may be as little as 6% of the maximum dose (Fig. 2A). However, even 6% of this dose (0.21 µM ODN:0.80 µM Pam2) is well within the antiviral range. To avoid presenting responses that are easily detectable but not physiologically relevant *in vivo*, all subsequent figures present data achieved with 0.21 µM ODN and 0.80 µM Pam2, except when the presented data are dose-response plots.

10.1128/mBio.00696-18.2 FIG S2  Higher dosing of Pam2-ODN further increases the antiviral effects on stimulated lung epithelial cells. HBEC3kt (A) or MLE-15 (B) cells were treated for 4 h with PBS (sham), combined treatment with 3.1 µM ODN and 12.4 µM Pam2, or combined treatment with 9.3 µM ODN and 37.2 µM Pam2, and then infected with influenza A virus at an MOI of 0.1. Results shown are expression levels of the viral NP gene relative to host 18S 24 h after infection. Data are representative of at least three independent experiments. *, *P* < 0.004 versus PBS-treated control; **, *P* < 0.02 versus 1× Pam2-ODN-treated control. Download FIG S2, EPS file, 0.8 MB. Copyright © 2018 Kirkpatrick et al. 2018 Kirkpatrick et al. This content is distributed under the terms of the Creative Commons Attribution 4.0 International license.

### Synergistic, durable, and rapid protection.

Concurrent treatment with Pam2 and ODN promotes greater survival of influenza virus in pneumonia than the additive effects of the two ligands delivered in isolation (10, 13). This was recapitulated in lung epithelial cells, where Pam2-ODN combination treatment resulted in substantial viral burden reductions while treatment with the individual ligands had no significant effect on viral gene expression (Fig. 2B). Also recapitulating our *in vivo* observations, treatment of lung epithelial cells with Pam2-ODN induced antiviral responses over a broad temporal range, including when treatment was delivered after the infection (Fig. 2C). Relatedly, the Pam2-ODN-induced viral burden benefit was evident very early after infection (Fig. 2D).

Viral M1 protein burden in lung epithelial cells infected with influenza A virus for 24 h revealed that Pam2-ODN reduced both the number of cells demonstrably infected with influenza virus and the per-cell viral protein amount among infected cells, resulting in a >90% reduction in virus burden (Fig. 2E and F). Comparable viral burden reductions were also observed when we assayed responses via immunoblotting for viral protein (Fig. 2G) or assessing tissue infectivity (Fig. 2H). Proportionate protection was achieved across a very broad range of influenza A virus inocula (Fig. 2I) and extended to lung epithelial cells challenged with Sendai virus (Fig. 2J). This effect does not appear to result from Pam2-ODN-induced changes in expression or distribution of sialic acid and its impact on viral attachment (Fig. S3). Effectively identical observations of Pam2-ODN-induced antiviral efficacy were made in experiments with murine MLE-15 lung epithelial cells and human A459 cells (Fig. S4).

10.1128/mBio.00696-18.3 FIG S3  Pam2-ODN does not modulate sialic acid-mediated virus attachment. (A) HBEC3kt lung epithelial cells were treated with Pam2-ODN or PBS for 4 h and then exposed to Cy3-conjugated Smabucus nigra (SNA) lectin to detect sialic acid and DAPI to label nuclei. Results shown are immunofluorescence micrographs (magnification, ×40; bar = 50 µm) (left) and mean fluorescence intensities per cell (right). (B) Epithelial cells were treated for 4 h with Pam2-ODN or PBS, and proteins were extracted and submitted to gel electrophoresis. The membranes were blotted with biotinylated SNA lectin and then exposed to Cy3-conjugated streptavidin. (C) Epithelial cells were treated for 4 h with Pam2-ODN or PBS, and then the cells were treated with Cy3-conjugated SNA lectin for 1 h. Protein was then extracted and submitted to gel electrophoresis. GAPDH loading controls for experiments shown in panels B and C are from the same membranes, but chemiluminescence was detected. (D) Epithelial cells were treated for 4 h with Pam2-ODN or PBS at 37°C and then infected on ice with biotinylated influenza A virus for 1 h. Cells were treated with sodium azide, mobilized, formalin fixed, exposed to Cy3-conjugated streptavidin, and submitted to flow cytometry to detect Cy3-positive cells. (E) Other cells were identically handled, except they were infected with nonbiotinylated virus and were probed with fluorescein isothiocyanate (FITC).-labeled anti-NP antibody. Download FIG S3, EPS file, 2.1 MB. Copyright © 2018 Kirkpatrick et al. 2018 Kirkpatrick et al. This content is distributed under the terms of the Creative Commons Attribution 4.0 International license.

10.1128/mBio.00696-18.4 FIG S4  Pam2-ODN induces antiviral responses from mouse and human lung epithelial cell lines. (A) MLE-15 cells were treated with PBS (sham) or escalating doses of Pam2-ODN for 4 h before infection with influenza A virus. Results shown are expression levels of viral NP gene relative to host 18S 24 h after infection. (B) MLE-15 cells were treated with PBS or Pam2-ODN at the indicated times relative to infection. Results shown are relative expression levels of viral NP gene at 24 h. (C) MLE-15 cells were treated with PBS or Pam2-ODN 4 h before infection with Sendai virus. Results shown are expression levels of M gene relative to host 18S at 24 h. Data are representative of at least three independent experiments. *, *P* < 0.005 versus PBS-treated control. Download FIG S4, EPS file, 2.8 MB. Copyright © 2018 Kirkpatrick et al. 2018 Kirkpatrick et al. This content is distributed under the terms of the Creative Commons Attribution 4.0 International license.

### Pam2-ODN promotes epithelial cell survival without reliance on type I interferon induction.

**FIG 3:**
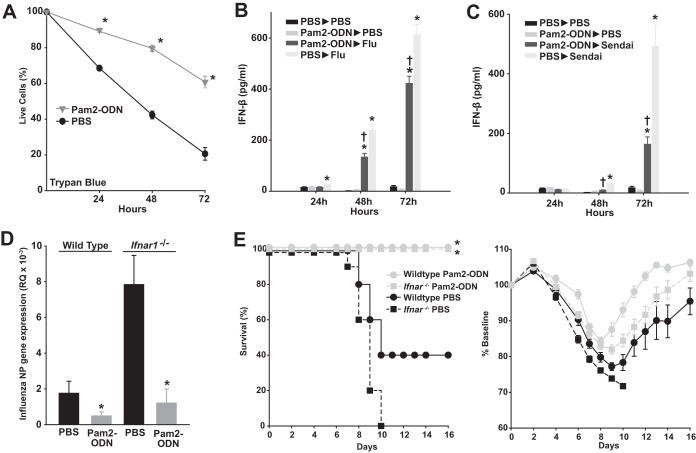
Pam2-ODN enhances epithelial cell survival after influenza virus infection without reliance on type I interferons. (A) HBEC3kt cells were treated for 4 h with Pam2-ODN or PBS and then infected with influenza A virus. At the indicated time points, the percentage of live cells was determined by trypan blue exclusion. (B and C) Primary mouse tracheal epithelial cells were grown at the air-liquid interface, treated with Pam2-ODN or PBS, and then infected with the indicated virus (or not). Results shown are interferon-β concentrations, measured by ELISA, at the indicated time points. (D) Influenza virus NP gene expression in primary tracheal epithelial cells of the indicated genotype 24 h after influenza virus infection following 4 h of treatment with PBS or Pam2-ODN. (E) Mice of the indicated genotype were treated with Pam2-ODN or PBS 24 h before challenge with influenza A virus. Results shown are survival (left) and weights (right). *, *P* < 0.05 versus isogenic PBS-treated samples; †, *P* < 0.02 versus PBS-treated/virus-infected samples.

Although Pam2-ODN reduced viral burdens, this did not address whether the response actually protects the host cells or whether there is any fitness cost to responding cells. Using trypan blue exclusion to differentiate live from dead cells, we found the survival of HBEC3kt cells was significantly increased by Pam2-ODN treatment at all time points following influenza virus infection (Fig. 3A). Most reported cell-protective antiviral responses are dependent on induction of type I interferon signaling (16). However, although type I interferons are induced by infection with influenza A virus or Sendai virus, we did not detect any increase in type I interferon concentrations following Pam2-ODN treatment of primary mouse tracheal epithelial cells (Fig. 3B and C). This is consistent with prior transcriptional analyses that revealed no enrichment of type I interferon genes or interferon-stimulated genes following Pam2-ODN treatment of mouse lungs or isolated lung epithelial cells (13–15), suggesting that interferon-independent mechanisms contribute to the inducible reductions in viral burden and enhanced survival. Indeed, while baseline susceptibility of interferon signaling-deficient primary tracheal epithelial cells was dramatically increased, Pam2-ODN still induced profound reductions in viral burden (Fig. 3D). Similarly, while sham-treated Ifnar1^−/−^ mice were more susceptible to influenza virus, they could be fully protected by Pam2-ODN (Fig. 3E).

**FIG 4:**
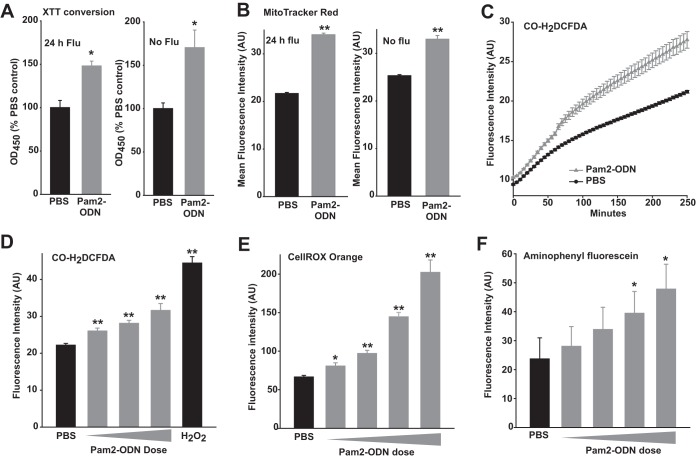
Pam2-ODN induces epithelial ROS production. (A) HBEC3kt cells were treated for 4 h with Pam2-ODN or PBS and then infected with influenza A virus for 24 h (left) or no virus (right). Results shown are the optical density at 450 nm (OD_450_) values 2 h after XTT exposure, normalized to OD_450_ values for PBS-treated cells. (B) HBEC3kt cells were treated for 4 h with Pam2-ODN or PBS, infected with influenza A for 24 h (left) or not (right), and then exposed to MitoTracker Red CMXRos. (C) Mean fluorescent intensities of HBEC3kt cells treated with PBS or Pam2-ODN after exposure to CO-H2DCFDA. (D) HBEC3kt cells treated with PBS, escalating doses of Pam2-ODN, or 100 µM H_2_O_2_ (positive control) after exposure to CO-H_2_DCFDA. (E) HBEC3kt cells treated with PBS or escalating doses of Pam2-ODN after exposure to CellROX orange. (F) HBEC3kt cells treated with PBS or escalating doses of Pam2-ODN after exposure to aminophenyl fluorescein. In panels C to F, fluorescence intensities represent results for eight samples per condition, reported 100 min after Pam2-ODN treatment. *, *P* < 0.005 versus isogenic PBS; **, *P* < 0.0005 versus PBS.

In parallel with the trypan blue exclusion assays, we performed experiments using 2,3bis-(2-methoxy-4-nitro-5-sulfophenyl)-2H-tetrazolium-5-carboxanilide salt (XTT) conversion and MitoTracker Red CMXRos fluorescence as indicators of cell viability. Congruent with the trypan blue data, Pam2-ODN pretreatment resulted in greater conversion of XTT and greater MitoTracker Red CMXRos fluorescence in HBEC3kt cells 24 h after infection with influenza A virus (Fig. 4A and B). While it might have been otherwise surmised that these findings were fully explained by enhanced survival of virus-challenged epithelial cells, we were surprised to find that Pam2-ODN also increased XTT conversion and MitoTracker Red CMXRos signal in the absence of viral challenge. While widely used as cell viability and proliferation markers, conversion of tetrazolium dyes such as XTT can also indicate mitochondrial activation (21) or ROS generation (22). Similarly, MitoTracker Red CMXRos fluorescence formally indicates the mitochondrial membrane potential (ΔΨ_m_), from which cellular viability is inferred. Thus, the signal enhancements in the absence of infection likely reflect a Pam2-ODN influence on ROS production and/or ΔΨ_m_.

### Induced epithelial ROS production.

Given the XTT assay results, we sought to characterize epithelial ROS generation following Pam2-ODN treatment. As most available reagents for detecting reactive oxygen and nitrogen intermediates have technical limitations related to species specificity and interactions with the species they measure (23–26), we assessed the induction of volatile species by comparing the detection patterns of an array of partially overlapping detectors. Pam2-ODN treatment of HBEC3kt cells in the presence of cell-permeant carboxy 2′,7′-dichlorodihydrofluorescein diacetate (CO-H_2_DCFDA) stimulated rapid, significant induction of fluorescent signal that was further separated from that of sham-treated samples throughout the period of observation (Fig. 4C). Dose-dependent ROS induction was observed in Pam2-ODN-treated cells whether they were exposed to CO-H_2_DCFDA, CellROX orange, or 3′-(*p*-aminophenyl) fluorescein (APF). To simplify visual comparisons, volatile species detection experimental results are presented as the mean fluorescence intensity 100 min after Pam2-ODN treatment (Fig. 4D to F).

Conversely, fluorescence was only induced from cells stained with 4-amino-5-methylamino-2′,7′-difluorescein (DAF-FM) diacetate at very high Pam2-ODN doses and was not induced at all with conventional DAF-FM (Fig. S5A and B), suggesting little induction of nitric oxide (NO) production. Further, there was no detectable induction of singlet oxygen or peroxynitrite at any Pam2-ODN dose (Fig. S5C and D). Together, these data suggest that Pam2-ODN induces epithelial production of superoxide (O_2_•^−^) and hydrogen peroxide (H_2_O_2_), and possibly hydroxyl radical (•OH).

10.1128/mBio.00696-18.5 FIG S5  Limited induction of epithelial reactive nitrogen species, singlet oxygen, or peroxynitrite by Pam2-ODN treatment. HBEC3kt cells were exposed to DAF-FM (A), DAF-FM diacetate (B), singlet oxygen Sensor Green (C), or dihydrorhodamine 123 (D) prior to treatment with PBS (sham) or escalating doses of Pam2-ODN. Results shown are fluorescence intensities 100 min after treatment. All panels are representative of at least three independent experiments. *n* = 8 wells/condition. *, *P* < 0.05 versus PBS-treated controls. Download FIG S5, EPS file, 2.2 MB. Copyright © 2018 Kirkpatrick et al. 2018 Kirkpatrick et al. This content is distributed under the terms of the Creative Commons Attribution 4.0 International license.

#### Antiviral protection requires ROS.

**FIG 5:**
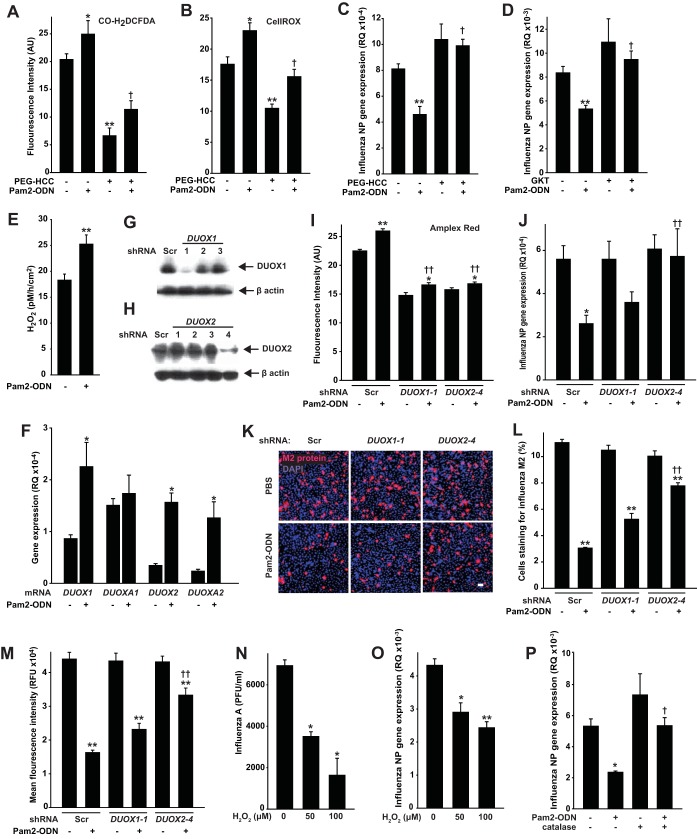
ROS generation is required for Pam2-ODN-induced epithelial antiviral responses. (A and B) HBEC3kt cells were pretreated with PEG-HCC or PBS. Results shown are the fluorescence intensity 100 min after treatment with PBS or Pam2-ODN following exposure to CO-H_2_DCFDA (A) or CellROX (B). (C) HBEC3kt cells were pretreated with PEG-HCC (or not), treated for 4 h with PBS or Pam2-ODN, and then infected with influenza A virus. Results shown are influenza A virus NP gene expression levels relative to host expression of 18S gene (RQ) 24 h after infection. (D) HBEC3kt cells were pretreated with the NADPH oxidase inhibitor GKT137831 or PBS, treated with PBS or Pam2-ODN, and then infected with influenza A virus. Results shown are relative influenza virus NP gene expression levels 24 h after infection. (E) Amplex red was used to determine H_2_O_2_ production in HBEC3kt cell-conditioned medium. (F) HBEC3kt cell expression of the indicated DUOX-related genes relative to 18S (RQ) 2 h after treatment with PBS or Pam2-ODN. (G and H) Immunoblots of HBEC3kt cells transfected with scrambled siRNA (Scr) or siRNA targeting *DUOX1* (G) or *DUOX2* (H). (I) Amplex red fluorescence 100 min after treatment of transfected cells with PBS or Pam2-ODN. (J and K) Transfected cells were treated for 4 h with PBS or Pam2-ODN, infected with influenza A virus, and then assessed for relative influenza virus NP gene expression (J) or immunofluorescent labeling for viral M2 protein 24 h after infection. Magnification (panel K), ×10. Scale bar = 50 µm. (L and M) Images in panel K were analyzed for M2-positive cells (L) and mean fluorescence per 1,000 cells (M). (N) Influenza A virus stock was exposed to H_2_O_2_ for 10 min, treated with catalase, inoculated into MDCK cell cultures, and submitted to hemagglutination assay. (O) HBEC3kt cells were exposed to H_2_O_2_ for 10 min, treated with catalase, and then challenged with influenza A virus. Results shown are NP gene expression levels 24 h after challenge. (P) HBEC3kt cells were treated with Pam2-ODN and/or catalase prior to influenza A virus challenge. *, *P* < 0.05 versus syngeneic PBS; **, *P* < 0.0005 versus syngeneic PBS; †, *P* < 0.0006 versus Pam2-ODN-treated samples without ROS scavenger/inhibitor pretreatment; ††, *P* < 0.05 versus Pam2-ODN-treated samples with scrambled siRNA.

To test the epithelial ROS requirement in therapeutically induced antiviral responses, we added polyethylene glycolated hydrophilic carbon clusters (PEG-HCCs) (27, 28) to culture media. Acting by superoxide dismutation and radical annihilation (27, 29), PEG-HCCs significantly reduced epithelial CO-H_2_DCFDA and CellROX fluorescence without exerting an intrinsic antiviral effect (Fig. 5A and B). The ROS-attenuating PEG-HCC treatment significantly impaired the Pam2-ODN-induced epithelial antiviral effect (Fig. 5C), supporting an epithelial ROS requirement for the protective response.

Similar to phagocytes, NADPH oxidases are the enzymes principally responsible for production of epithelial ROS (30, 31). To ensure that the effects of PEG-HCC were exerted via ROS-dependent mechanisms and to begin to identify relevant ROS sources, we tested epithelial cells in the presence of the NADPH oxidase inhibitor GKT137831. By attenuating inducible production of NADPH oxidase-dependent ROS, we confirmed a recent report (32) that NADPH oxidase inhibition increases baseline epithelial susceptibility to influenza virus, and we discovered that NADPH-dependent ROS production is also required for Pam2-ODN-induced antiviral protection (Fig. 5D). Although multiple NADPH oxidase isoforms (NOX) are expressed in lung epithelia, the primary sources of ROS are the dual oxidases DUOX1 and DUOX2 (also called NOX6 and NOX7) (30–32). Amplex Red testing of conditioned media revealed that Pam2-ODN treatment of HBEC3kt cells stimulated production of H_2_O_2_ (Fig. 5E), the primary product of DUOX1 and DUOX2. Moreover, gene expression analyses revealed enrichment of *DUOX1*, *DUOX2*, and *DUOXA2* following Pam2-ODN treatment, with *DUOXA1* expression relatively high at baseline (Fig. 5F).

To test the requirement for DUOX-derived ROS in the Pam2-ODN-induced antiviral defense, we used shRNA to stably knock down *DUOX1* and *DUOX2* in HBEC3kt cells (Fig. 5G and H). Knocking down either DUOX gene substantially reduced the H_2_O_2_ produced by cells, though a small but significant increase was still observable following Pam2-ODN treatment (Fig. 5I). When we tested the functional importance of the H_2_O_2_ impairment, we found that knocking down *DUOX2* profoundly impaired Pam2-ODN-inducible protection, while knocking down *DUOX1* appeared to have a more modest effect (Fig. 5J to M). Loss of protection in the ROS-impaired cells did not appear to arise from direct epithelial toxicity of the inhibitors or scavengers (Fig. S6). Application of H_2_O_2_ directly to virus before scavenging with catalase impaired viral infectivity, and applying H_2_O_2_ to HBEC3kt cells before scavenging with catalase reduced the influenza virus burden 24 h after challenge (Fig. 5N to P), suggesting that ROS may play both direct virucidal and also signaling roles in protection.

10.1128/mBio.00696-18.6 FIG S6  ROS inhibition and scavenging strategies have no detected effect on cell survival. HBEC3kt cells deficient in dual oxidase genes (A) or exposed to ROS scavengers (B) were submitted to trypan blue exclusion assays to determine the number of viable cells in culture. No significant intergroup differences were detected. Download FIG S6, EPS file, 2 MB. Copyright © 2018 Kirkpatrick et al. 2018 Kirkpatrick et al. This content is distributed under the terms of the Creative Commons Attribution 4.0 International license.

#### Epithelial antiviral responses require mitochondrial ROS.

**FIG 6:**
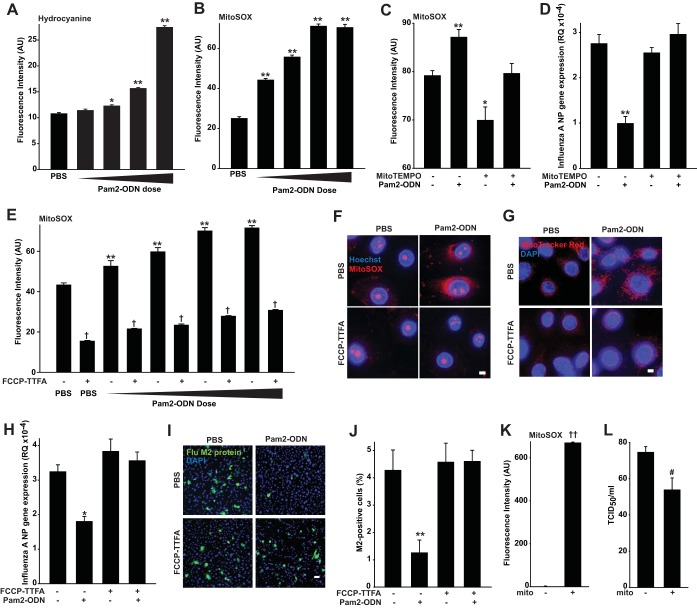
Pam2-ODN-induced protection is dependent upon mitochondrial ROS. (A and B) HBEC3kt cells were treated with PBS or escalating doses of Pam2-ODN after exposure to hydrocyanine (A) or MitoSOX (B). (C) MitoSOX fluorescence of HBEC3kt cells pretreated (or not) with MitoTEMPO and then treated with PBS or Pam2-ODN. (D) HBEC3kt cells were pretreated with MitoTEMPO (or not), treated with PBS or Pam2-ODN, and then infected with influenza A virus. Results shown are the expression levels of influenza A virus NP 24 h after infection. (E) MitoSOX fluorescence of HBEC3kt cells exposed to PBS or escalating doses of Pam2-ODN in the presence or absence of mitochondrial electron chain inhibitors. (F and G) HBEC3kt cells were pretreated with mitochondrial electron chain inhibitors (or not), treated with PBS or Pam2-ODN, and then imaged using MitoSOX (magnification, ×100; bar = 5 µm) (F) or MitoTracker Red CMXRos (magnification, ×100; bar = 50 µm) (G). (H) HBEC3kt cells were pretreated with mitochondrial electron chain inhibitors (or not), treated with PBS or Pam2-ODN, and then infected with influenza A virus. Results shown are relative influenza virus NP gene expression levels 24 h after infection. (I) HBEC3kt cells were pretreated with mitochondrial electron chain inhibitors (or not), treated with PBS or Pam2-ODN, and then infected with influenza A virus. Results shown are merged images of DAPI and fluorescently labeled M2 antibody (magnification, ×10; bar = 50 µm). (J) Percentage of M2-positive cells in panel I. (K) MitoSOX detection of freshly isolated mitochondria from lung homogenates versus medium alone. (L) Hemagglutination assay results for influenza virus inocula with or without 15-min exposure to isolated mitochondria in panel K. *, *P* < 0.005 compared to PBS-treated control; **, *P* < 0.0005 compared to PBS-treated control; †, *P* < 0.001 compared to the same Pam2-ODN dose but without inhibitor; ††, *P* < 0.0001 compared to no-mitochondria control; #, *P* = 0.01 compared to no-mitochondria control.

That Pam2-ODN enhanced ΔΨ_m_ prompted us to hypothesize that mitochondrial ROS (mtROS) might also contribute to protection, in addition to DUOX-derived ROS. Further, PEG-HCC functioning partly by superoxide dismutation also suggested that non-H_2_O_2_ species (hence, from non-DUOX sources) also contributed to the protection. Indeed, we found that Pam2-ODN also induced dose-dependent generation of hydrocyanine-detected superoxide, the principal species produced by mitochondria (Fig. 6A). Then, we used MitoSOX Red as a targeted marker of mtROS concentration to further support that Pam2-ODN induces dose-dependent increases in mtROS (Fig. 6B). The mitochondrion-targeted superoxide dismutase mimetic mitoTEMPO reduced the Pam2-ODN-induced MitoSOX Red signal to baseline levels (Fig. 6C). This completely abrogated the protective antiviral response and revealed a requirement for mtROS in Pam2-ODN-induced protection (Fig. 6D).

**FIG 7:**
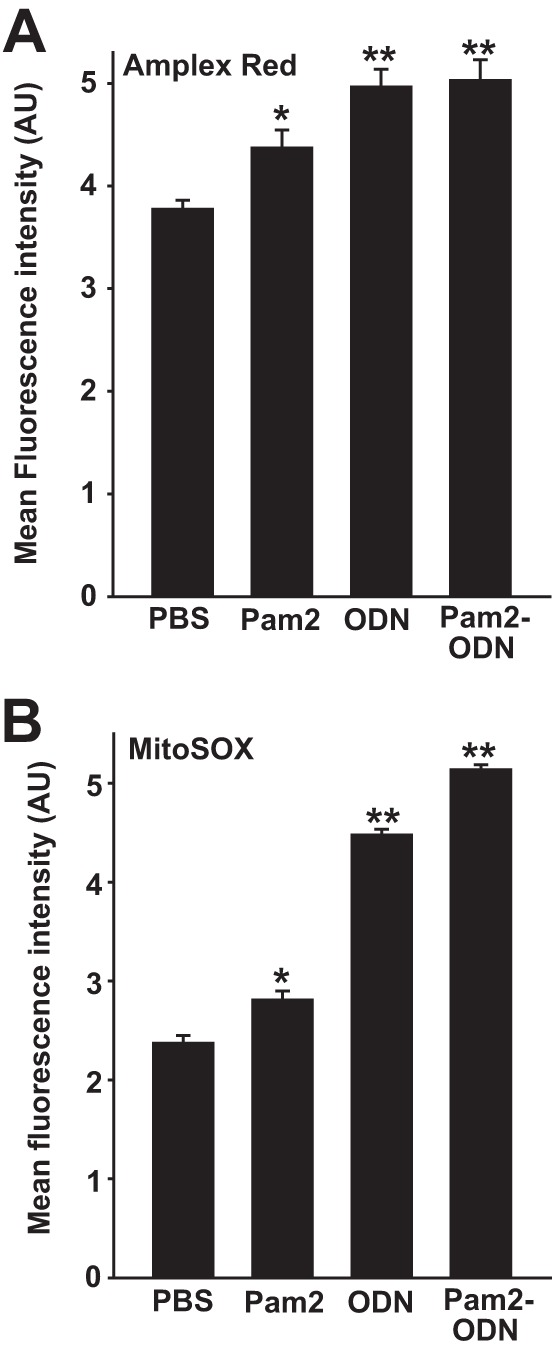
Pam2 and ODN induce cellular and mitochondrial ROS. HBEC3kt cells were treated with the indicated agents after exposure to Amplex Red (A) or MitoSOX (B). Results shown are fluorescence intensities 100 min after treatment. *, *P* < 0.01 compared to PBS-treated control; **, *P* < 0.008 compared to PBS-treated control.

To ensure that the mitoTEMPO effect was not attributable to nonspecific scavenging of nonmitochondrial ROS, we tested whether pharmacological blockade of inducible mtROS would attenuate antiviral resistance. Although mitochondrial electron transport chain modulators have unpredictable, often opposing, effects on ΔΨ_m_ and mtROS in different models, we found that simultaneous application of trifluoromethoxy carbonylcyanide phenylhydrazone (FCCP), an uncoupler of oxidative phosphorylation, and 2-thenoyltrifluoroacetone (TTFA), a complex II inhibitor, reliably reduced both baseline and Pam2-ODN-induced mtROS production and ΔΨ_m_ (Fig. 6E to G). As with mtROS scavenging, FCCP-TTFA inhibition of Pam2-ODN-induced mtROS production completely abrogated the antiviral effect (Fig. 6H to J). Congruently, transiently exposing influenza virus to actively respiring mitochondria isolated from mouse lungs impaired viral infectivity (Fig. 6K and L). While Pam2 and ODN can each individually induce significant DUOX and mitochondrial ROS generation, it appears that ODN is the stronger stimulus of ROS production from both sources (Fig. 7A and B), offering insight into how ODN contributes to the protection afforded by Pam2.

#### Inducible mtROS are required for antiviral protection.

**FIG 8:**
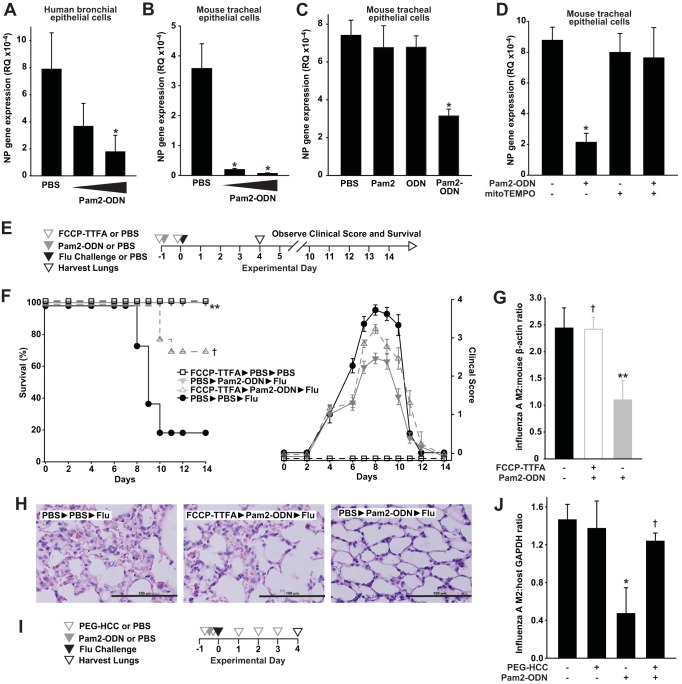
Pam2-ODN induces mtROS-dependent antiviral responses from primary lung epithelial cells and intact lungs. (A and B) Primary human bronchial epithelial cells (A) or mouse tracheal epithelial cells (B) were cultured in differentiation medium under air-liquid interface conditions, treated with PBS or Pam2-ODN for 4 h, infected with influenza A virus, and then harvested after 24 h. Results are the expression levels of the influenza A virus nucleoprotein (NP) gene. (C) Primary mouse tracheal epithelial cells were treated with PBS, Pam2, ODN, or both ligands prior to infection with influenza A virus. Results shown are the relative expression levels of the influenza virus NP gene 24 h after infection. (D) Primary mouse tracheal epithelial cells were pretreated with MitoTEMPO (or not), treated for 4 h with PBS or Pam2-ODN, and then infected with influenza A virus. Results shown are the relative expression levels of the influenza virus NP gene 24 h after infection. (E) Schematic of *in vivo* challenge. (F) Survival (left) and clinical scores (right) of mice treated with mtROS inhibitors and/or Pam2-ODN prior to infection (or not) with influenza virus. (G) Quantification of lung viral burdens of mice in panel F by immunoblotting for influenza virus M2 protein 4 days after infection. (H) Hematoxylin and eosin-stained micrographs of lungs from the indicated treatment groups. Magnification, ×40. Bar = 100 µm. Mice were treated with PEG-HCC or PBS daily from the day before infection until lung harvest. (I) Treatment schedule. (J) Influenza virus burden 4 days after infection. *, *P* < 0.008 compared to PBS-treated control; **, *P* < 0.0001 compared to PBS-treated and infected controls; †, *P* < 0.04 compared to Pam2-ODN-treated group that received no ROS inhibitor/scavenger.

To ensure that the responses observed in mouse and human lung epithelial cell lines were representative of native responses, viral protection studies were repeated in primary human and mouse lung epithelial cells grown at the air-liquid interface. Again, we observed dose-dependent inductions of epithelial antiviral responses by Pam2-ODN (Fig. 8A and B) that revealed synergistic ligand interactions (Fig. 8C). As with the immortalized cell lines, the antiviral protection was completely abolished by mtROS scavenging (Fig. 8D).

Testing the *in vivo* requirement for mtROS in the Pam2-ODN-induced defense, mice were treated with FCCP-TTFA by aerosol to block lung epithelial mtROS induction. To avoid effects on mucociliary virus clearance, inhibitor treatments were given before and after Pam2-ODN treatments, but not after viral challenge. mtROS inhibition significantly attenuated the Pam2-ODN-induced survival advantage, and the clinical scores of surviving mice revealed considerably greater morbidity than among those that received Pam2-ODN without mtROS inhibition (Fig. 8E and F). No toxicity was observed in uninfected FCCP-TTFA-treated mice. Concordant with the effects on survival and clinical score, mice that received mtROS inhibitors displayed significantly higher viral lung burdens than the mice that received Pam2-ODN only (Fig. 8G), and their lungs demonstrated more immunopathology (Fig. 8H). Unlike the *in vitro* study findings, it is possible that the mtROS inhibitors also exert effects on nonepithelial cells or that epithelial mtROS impairment may alter epithelial signals to other contributing cells, and this possibility will be investigated. Scavenging of cellular ROS by PEG-HCC also impaired Pam2-ODN-induced viral killing (Fig. 8I and J), though continuous treatment with a scavenger over the extended period of observation was not practical, precluding assessments of survival.

## DISCUSSION

Although the airway and alveolar epithelia are often regarded as passive airflow conduits or inert gas exchange barriers, it is evident that they possess intrinsic antimicrobial capacities that contribute to pathogen clearance under physiological conditions (17, 33–35). The current work further substantiates the role of the lung epithelium in the therapeutic induction of antiviral resistance and establishes the sufficiency of isolated epithelial cells to generate protective antiviral responses following Pam2-ODN treatment. While challenging paradigms about the role of the epithelium in antiviral defense, these findings also offer the potential to protect patients from viral respiratory infections during periods of vulnerability.

Viral respiratory tract infections are extremely common causes of morbidity. More than 200 viruses cause respiratory infections (36). These diverse threats demonstrate a need for broadly protective antiviral strategies that can be applied prior to pathogen identification. Here, we demonstrated robust protection against an orthomyxovirus and a paramyxovirus, whether Pam2-ODN was delivered before or after infection.

As epithelial cells are principal targets of respiratory viruses (16), it is conceptually appealing to directly stimulate them to avoid establishment and progression of viral infections. Further, since respiratory viral infections frequently exacerbate preexisting lung diseases (37) and since epithelial immune functions are central to both respiratory virus clearance and asthma pathogenesis (36, 38–40), broadly stimulating epithelial antiviral responses could conceivably improve health outcomes both by reducing acute respiratory infection rates and by enhancing control of preexisting lung disease.

Single-stranded RNA viruses are primarily detected by RIG-I-like receptors, but TLR functions also contribute to responses to influenza virus infections (41). However, while most protective antiviral responses are tightly linked to interferon-stimulated genes (37, 41), we did not detect type I interferon responses to Pam2-ODN treatment, and we observed no impaired protection in the absence of type I interferon signaling. Thus, we sought interferon-independent antiviral mediators to explain the robust protection, and we found ROS to be essential mediators.

ROS detection in the lungs is inherently challenging. Direct observation of most species is only possible on a submillisecond time scale, and the reagents available for ROS detection lack specificity (24). However, dose-dependent induction of signal from multiple detectors following Pam2-ODN treatment of epithelial cells is strongly supportive of ROS induction (23), as is the loss of protection when ROS generation is impaired by pharmacological or genetic means. Superoxide and H_2_O_2_ are the predominant species produced by lung cells (25, 42, 43), and the described spectra of the tested ROS detectors suggest these are the primary ROS induced by Pam2-ODN treatment. This is consistent with the demonstrated requirement for DUOX2 and mitochondrial ROS production for protection. Conversely, we detected little increase in NO levels following Pam2-ODN treatment. This is notable, since increased levels of inducible nitric oxide synthase and NO have been reported to contribute to antiviral epithelial responses to rhinoviruses (37).

Antibacterial roles for ROS have been increasingly described, and ROS appear to interact favorably with other antimicrobial molecules, such as neutrophil proteases (43). The role of ROS in antiviral defense is much less clear. Some reports describe ROS-associated oxidative stress as an influenza virus virulence factor (44), while others find that influenza virus infections lead to decreased ROS production (32). These seemingly contradictory observations underscore the complex management of oxidant balance in airways (45) and, by corollary, support the relevance of such strong induction of ROS by Pam2-ODN.

Impairment of DUOX enzymes in bronchial (32) or nasal (46) epithelial cells permits increased viral replication, and the DUOX2-dependent product of the lactoperoxidase/H_2_O_2_/thiocyanate system, hypothiocyanate, exerts virucidal effects (47). Further, we and others have observed induction of DUOX-related genes in a number of models (31, 48, 49). Thus, our finding of DUOX2 participation in Pam2-ODN-induced protection is not entirely surprising. However, it is unlikely that the ROS dependency simply reflects H_2_O_2_-mediated hypothiocyanate production, as our *in vitro* models lack tracheobronchial seromucus glands as a lactoperoxidase source (50) and our models lack a source of thiocyanate (48). Moreover, Pam2-ODN-induced protection is achieved without applying ATP or manipulating calcium concentrations to stimulate ROS production from DUOX2, as applied in prior antiviral models (51, 52). The precise mechanisms of the inducible DUOX2-mediated virucidal activity remains an area of active investigation.

Unlike NOX/DUOX-generated ROS, mtROS are generated via leakage from the electron transport chain (25), resulting in production of superoxide that diffuses through mitochondrial membranes once dismutated to H_2_O_2_ (24). This process is exquisitely tightly regulated by changes in scavenging, production, and localization (42, 53), so robust induction of mtROS by Pam2-ODN represents a notable homeostatic perturbation.

mtROS are being increasingly described to participate in innate and adaptive immunity (42). TLR-initiated signaling can induce antibacterial mtROS production in macrophages (54), and antibacterial effects of coregulated NOX2- and mitochondrion-derived ROS have been described in phagocytes (55). However, protective coordinated ROS production has not been previously reported from these sources (DUOX2 and mitochondria), in the cells (lung epithelium), or against viruses. Scavenging of mtROS in nasal epithelial cells appears to increase baseline susceptibility to viral infection (56). However, these native antiviral responses require induction of interferon-sensitive genes that are not enriched by Pam2-ODN treatment. While working to understand how mitochondria contribute to an epithelial antiviral state, it is also interesting to consider whether some of the effects of mitochondrial inhibition may arise from the highly related issue of ΔΨ_m_ (57), rather than strictly from mtROS.

Taken together, these data reinforce the antimicrobial capacity of the lung epithelia and provide insights into how these native defense may be exploited to protect patients during periods of intensive virus exposure or when leukocyte elements of the immune response are impaired.

## MATERIALS AND METHODS

### Animals, cells, and reagents.

All general reagents were obtained from Sigma-Aldrich (St. Louis, MO), except as indicated. All mouse experiments were performed with 5- to 8-week-old C57BL/6J or Ifnar1^−/−^ mice (The Jackson Laboratory, Bar Harbor, ME) of a single gender, in accordance with the Institutional Animal Care and Use Committee of the University of Texas MD Anderson Cancer Center, protocol 00000907-RN01. Immortalized human bronchial epithelial (HBEC3kt) cells were kindly provided by John Minna. Murine lung epithelial (MLE-15) cells were kindly provided by Jeffrey Whitsett. Normal human bronchial epithelial (NHBE) cells were purchased from Lonza (Basel, Switzerland). Immortalized cells were authenticated by the MD Anderson Characterized Cell Line Core Facility. Mouse-adapted influenza A/Hong Kong/8/68 virus (H3N2; Mouse Lung Pool 1/17/12) was kindly provided by Brian E. Gilbert (58). Sendai virus (parainfluenza virus type 1) was obtained from the American Type Culture Collection (ATCC; Manassas, VA).

### Cell culture.

HBEC3kt cells were cultured in keratinocyte serum-free medium (KSFM; Thermo, Fisher Scientific, Grand Island, NY) supplemented with human epidermal growth factor and bovine pituitary extract. MLE-15 cells were cultured in RPMI supplemented with 10% fetal bovine serum. Cultures were maintained in the presence of penicillin and streptomycin. NHBE cells were expanded in submerged culture using a Clonetics B-ALI air-liquid interface protocol and reagents (Lonza) and then seeded into 24-well plates containing transwell inserts coated with rat tail collagen type 1 (BD Biosciences, East Rutherford, NJ). Three days after seeding, B-ALI growth medium was removed from both apical and basal chambers, and B-ALI differentiation medium was added to only the basal chambers.

### TLR treatments.

For *in vivo* studies, *S*-[2,3-bis(palmitoyloxy)-propyl]-(*R*)-cysteinyl-(lysyl) 3-lysine (Pam2CSK_4_) and ODN M362 (InvivoGen, San Diego, CA) were reconstituted in endotoxin-free water and then diluted to the desired concentration in sterile phosphate-buffered saline (PBS). As previously described (14), the Pam2-ODN was placed in an Aerotech II nebulizer (Biodex Medical Systems, Shirley, NY) driven by 10 liters min^−1^ air supplemented with 5% CO_2_ for 20 min. The nebulizer was connected by polyethylene tubing to a polyethylene exposure chamber. Twenty-four hours prior to infections, 8 ml of Pam2 (4 µM):ODN (1 µM) was delivered via nebulization to unrestrained mice for 20 min, and then mice were returned to normal housing. For *in vitro* studies, Pam2-ODN was added to the culture medium 4 h prior to inoculation with virus or at the indicated time point. Pam2-ODN was used in a fixed concentration ratio but at varying doses, as indicated in the figures and Results section.

### Infection models.

As previously described (14), frozen stock (2.8 × 10^7^ 50% tissue culture infective doses [TCID_50_] ml^−1^) of virus was diluted 1:250 in 0.05% gelatin in Eagle’s minimal essential medium and delivered by aerosolization for 20 min to achieve the 90% lethal dose (LD_90_) to LD_100_ (~100 TCID_50_ per mouse). Viral concentrations in the nebulizer before and after aerosolization were determined by hemagglutination assay of infected Madin-Darby canine kidney (MDCK) cells (ATCC, Manassas, VA). Sendai virus (parainfluenza virus type 1) was obtained from ATCC and expanded on cultures of rhesus monkey kidney (RMK) cells (ViroMed Laboratories, Minnetonka, MN) in LHC-8 medium (Thermo, Fisher). Following virus liberation and gradient purification, 40 µl of viral suspension (1.6 × 10^6^ PFU/mouse) in sterile PBS was delivered to anesthetized mice via intrapharyngeal instillation. For each survival group, *n* was 10 to 15 mice. Animals were weighed daily and sacrificed if they met euthanasia criteria, including signs of distress or loss of 25% preinfection body weight. To supplement the characterization of animal morbidity, clinical scores were assigned to all mice by an investigator (V.V.K.) who was blinded to group allocation. To calculate the clinical score, one point was assigned to a mouse for each of the observed changes of hunched posture, ruffled fur, and reduced mobility, resulting in a maximum score of three points per animal. Additional animals (*n* = 4 per group) were sacrificed 4 days after infection and lung homogenates were assayed for viral burden as described below. For *in vitro* infections, viral inocula (multiplicities of infection [MOI] of 0.01 to 1.0) of each strain were added to cells in submerged monolayer or air-liquid interface cultures, as indicated for each experiment.

### Pathogen burden quantification.

To measure transcript levels of influenza A virus nucleocapsid protein (*np1*) and Sendai matrix protein (*M*), samples were harvested in RNAlater (Qiagen, Hilden, Germany) and RNA was extracted using the RNeasy extraction kit (Qiagen). Five hundred nanograms of total RNA was reverse transcribed to cDNA by using an iScript cDNA synthesis kit (Bio-Rad, Hercules, CA) and submitted to quantitative reverse transcription-PCR (RT-PCR) analysis with SYBR green PCR master mix (Thermo, Fisher) on an ABI ViiA 7 real-time PCR system. Host 18S rRNA was similarly probed to determine relative expression of viral transcripts. To measure levels of viral protein, immunoblotting for influenza A virus M1 or M2 was performed, and results are presented relative to host β-actin levels. To measure tissue infectivity, serial dilutions of lysed samples were added to cultures of MDCK cells. After 4 days, 1% turkey red blood cell solution was added to cultures and TCID_50_ values were calculated based on hemagglutination.

### Indirect immunofluorescence assay.

Epithelial cells were grown on glass coverslips, treated as indicated in the Results section, fixed in 2% paraformaldehyde, permeabilized with 0.1% Triton X-100, and blocked with 10% serum in PBS. Cells were incubated with primary antibodies against viral M1 or M2 proteins (Santa Cruz Biotechnology) at a dilution of 1:200 for 1 h, then with AlexaFluor secondary antibodies (Life Technologies, Inc., Carlsbad, CA) at a dilution of 1:500 for half an hour, and counterstained with 4′,6-diamidino-2-phenylindole (DAPI). Cells were visualized using a DeltaVision deconvolution fluorescence microscope (GE Life Sciences). Fluorescence intensity was quantified using ImageJ.

### Cell viability assays.

Epithelial cell viability was determined by formazan complex formation using the colorimetric XTT cell viability kit (Cell Signaling, Inc., Danvers, MA) read *in situ* on a BioTek Synergy2 plate reader. For trypan blue exclusion assays, PBS-washed cells were mobilized with 0.25% trypsin, washed in PBS again, resuspended in 100 µl of PBS, and mixed with equal volumes of 0.4% trypan blue dye. Cell counts in samples were manually determined on a hemacytometer.

### Lentiviral shRNA knockdown of DUOX1 and DUOX2.

GIPZ human *DUOX1* and *DUOX2* lentiviral shRNA clones were purchased from GE Dharmarcon (Lafayette, CO). Lentiviruses bearing human *DUOX1* and *DUOX2* shRNA were produced by transfection in 293T cells per the manufacturer’s instructions. Infection efficiency was enhanced by addition of 8 µg/ml Polybrene into the culture medium and centrifuging the cells at 2,000 rpm for 60 min at 32°C. Lentivirus-infected HBEC3kt cells were selected by cell sorting based on GFP expression. shRNA knockdown efficiency was determined by immunoblot analysis.

### ROS detection, scavenging, and inhibition.

To assess ROS generation, cells were treated with 5 µM of each indicated detector for 1 h before exposure to Pam2-ODN or the sham treatment. Fluorescence was continuously measured on a BioTek Synergy2 for 250 min after treatment. Excitation/emission wavelengths for ROS-detecting agents are as follows: CO-H_2_DCFDA, 490 nm/525 nm; APF, 490 nm/525 nm; hydrocyanine (ROSstar 550; Li-Cor, Lincoln, NE), 510 nm/580 nm; 4-amino-5-methylamino-2′,7′-difluorofluorescein (DAF-FM and DAF-FM diacetate), 490 nm/525 nm; MitoSOX red, 510 nm/580 nm; MitoTracker Red CMXRos, 510 nm/580 nm; Amplex red, 570 nm/585 nm. The H_2_O_2_ concentration in conditioned medium was interpolated from a standard curve as previously reported (52).

Cellular ROS were scavenged by 1 h exposure to PEG-HCC (5 µg/ml) prior to application of Pam2-ODN or PBS. Mice were treated by nebulization of PEG-HCC (0.5 mg/ml) in 8 ml PBS 2 h before Pam2-ODN (or PBS-0 treatment) immediately after Pam2-ODN treatment and daily after influenza virus challenge. Mitochondrial ROS were scavenged by 1 h exposure to MitoTEMPO (30 nM; Thermo, Fisher) prior to treatment with Pam2-ODN or PBS. NADPH oxidase activity was inhibited by 1 h exposure to GKT137831 (10 µM; Selleckchem, Houston, TX). Disruption of *in vitro* mitochondrial ROS production was achieved through concurrent application of FCCP (400 nM, Cayman Chemical, Ann Arbor, MI), and TTFA (200 µM; Sigma). Mice were treated with 10 ml TTFA (200 mM) and FCCP (800 µM) by aerosol. Groups received TTFA-FCCP (or sham) 5 h before infection, Pam2-ODN (or sham) 4 h before infection, or TTFA-FCCP (or sham) 2 h before infection and then were submitted to influenza A virus challenge.

### Immunoblotting.

For immunoblot assays, samples were exposed to lysis buffer in the presence of Halt protease and phosphatase inhibitor cocktail (Millipore), and the protein concentration in lysate was determined in a bicinchoninic acid protein assay. Thirty-five micrograms of protein in 2× Laemmli buffer was separated by SDS-PAGE and then transferred onto polyvinylidene difluoride membranes. The blots were probed with influenza A virus M2 (14C2), DUOX1 (H-9; Santa Cruz Biotechnology), and DUOX2 (AP11227c; Abgent, San Diego, CA) primary antibodies, detected by secondary antibodies with conjugated horseradish peroxidase, and developed using a Pico-sensitive chemiluminescence kit (Pierce). All membranes were stripped and probed for β-actin as the loading control. Densitometric semiquantitation of the protein band intensities was performed using ImageJ.

### Mitochondrial isolation and challenge.

As previously described (59), mouse lungs were excised and then disrupted using a Polytron homogenizer (Pro Scientific, Oxford, CT). Mitochondria were extracted via serial centrifugation. The isolated mitochondria were plated in round-bottom 96-well plates, and active mitochondrial respiration was confirmed based on MitoSOX fluorescence. Thereafter, freshly thawed influenza virus was exposed to either the extracted mitochondria in Dulbecco’s modified Eagle’s medium or medium alone for 10 min, then serial dilutions of the influenza virus-containing medium was plated onto MDCK cells for a hemagglutination assay.

### Statistical analysis.

Statistical analysis was performed using SPSS v19 (SAS Institute, Cary, NC). Student’s *t* test was used to compare the lung viral burdens between the groups. Error bars shown in all the figures represent technical replicates within the displayed experiment, rather than aggregation of experimental replicates. Percentages of mice surviving pathogen challenges were compared using the Fisher exact test on the final day of observation, and the log rank test was used to compare the survival distribution estimated by the Kaplan-Meier method.
