# Erratum for Kirkpatrick et al., “Inducible Lung Epithelial Resistance Requires Multisource Reactive Oxygen Species Generation To Protect against Viral Infections”

**DOI:** 10.1128/mBio.00496-19

**Published:** 2019-04-02

**Authors:** Carson T. Kirkpatrick, Yongxing Wang, Miguel M. Leiva Juarez, Pooja Shivshankar, Jezreel Pantaleón García, Alexandria K. Plumer, Vikram V. Kulkarni, Hayden H. Ware, Fahad Gulraiz, Miguel A. Chavez Cavasos, Gabriela Martinez Zayas, Shradha Wali, Andrew P. Rice, Hongbing Liu, James M. Tour, William K. A. Sikkema, Ana S. Cruz Solbes, Keith A. Youker, Michael J. Tuvim, Burton F. Dickey, Scott E. Evans

**Affiliations:** a Department of Pulmonary Medicine, University of Texas MD Anderson Cancer Center, Houston, Texas, USA; b Tecnológico de Monterrey School of Medicine, Monterrey, Mexico; c The University of Texas Graduate School of Biomedical Sciences, Houston, Texas, USA; d Department of Molecular Virology and Microbiology, Baylor College of Medicine, Houston, Texas, USA; e Smalley Institute for Nanoscale Science and Technology, Rice University, Houston, Texas, USA; f Michael E. Debakey Heart and Vascular Institute, Houston Methodist Hospital, Houston, Texas, USA

## ERRATUM

Volume 9, issue 3, e00696-18, 2018, https://doi.org/10.1128/mBio.00696-18. In the published version of this article, the name of author Gabriela Martinez Zayas was misspelled. This erratum serves to correct the spelling.

